# Supplementary material for: Rapid binding to protofilament edge sites facilitates tip tracking of EB1 at growing microtubule plus-ends
Source: eLife. 2024 Feb 22;13:e91719. doi: 10.7554/eLife.91719 (PMC10883673; doi:10.7554/eLife.91719)
Supplement: Supplementary file 1. — Parameters determine the on and off rates of tubulin subunits from the microtubule tip, as well as parameters that control the hydrolysis rate of GTP-tubulin subunits within the lattice. [file elife-91719-supp1.docx]

Table S1: Simulation Parameters: Microtubule Assembly

| Parameter | Description | Value | Reference |
| --- | --- | --- | --- |
| [GTP-tub] | Free GTP-Tubulin concentration | 3-15 μM | Matched to experiments |
| *k_on, PF_* | Tubulin on-rate constant | 0.65 µM^-1^ s^-1^ pf^-1^ | (Margolin et al., 2012), lowered to match experimental growth rates (Roth et al., 2019) |
| *k_off, GTP_* | Tubulin off-rate when GTP dimer below | 0.2 s^-1^ | (Margolin et al., 2012) |
| *k_off, GDP_* | Tubulin off-rate when GDP dimer below | 200 s^-1^ | (Margolin et al., 2012) |
| *k_hyd, GTP_* | Hydrolysis rate constant | 0.55 s^-1^ | (Margolin et al., 2012) |
| *k_lateral Bond Formation_* | Formation rate for a lateral bond between protofilaments | 100 s^-1^ | (Margolin et al., 2012) |
| *k_lateral bond break TT_* | Breakage Rate for lateral bond between two GTP dimers | 70 s^-1^ | (Margolin et al., 2012) |
| *k_lateral bond break TD_* | Breakage rate for lateral bond between a GTP and GDP dimer | 90 s^-1^ | (Margolin et al., 2012) |
| *k_lateral bond break DD_* | Breakage rate for lateral bond between two GDP dimers | 600 s^-1^ | (Margolin et al., 2012) |
| *k_lateral bond break TT Seam_* | Breakage Rate for lateral bond two GTP dimers at the seam | 140 s^-1^ | (Margolin et al., 2012) |
| *k_lateral bond break TD Seam_* | Breakage Rate for lateral bond between a GTP and GDP dimer at the seam | 180 s^-1^ | (Margolin et al., 2012) |
| *k_lateral bond break DD Seam_* | Breakage Rate for lateral bond between two GDP dimers at the seam | 1200 s^-1^ | (Margolin et al., 2012) |
| *π_break_* | Correction factor for the lateral bond breakage rate if the neighboring PFs all have lateral bonds | 10 | (Margolin et al., 2012) |
| *T­_dimer_* | Max taper length in dimers before increase in k_lateral bond break_ and k_off_ | 75 layers (600 nm) | Based on (Ogren et al., 2022) |
| *π­_taperOff_* | Factor to increase off rates when taper greater than T_dimer_ | 100 | this study |
| *π­_taperLateralBreak_* | Factor to increase lateral bond breaking rates when taper greater than T_dimer_ | 1000 | this study |
