# Supplementary material for: Rapid binding to protofilament edge sites facilitates tip tracking of EB1 at growing microtubule plus-ends
Source: eLife. 2024 Feb 22;13:e91719. doi: 10.7554/eLife.91719 (PMC10883673; doi:10.7554/eLife.91719)
Supplement: Supplementary file 2. — Parameters determine the on and off rates of EB1 molecules from the microtubule tip and lattice. [file elife-91719-supp2.docx]

Table S2: Simulation Parameters: EB1 Dynamics

| Parameter | Description | Simulation Baseline Value | Reported Literature Values | Reference |
| --- | --- | --- | --- | --- |
| [EB1] | Free EB1 concentration in the simulation | 200 nM | N/A | Matched to Experiment |
| *k_on, Lattice_* | EB1 on-rate constant per closed-lattice binding site  (GTP or GDP) | ~0.09-0.18 s^-1^ nM^-1^  *(for comparison purposes, this value was scaled up from 2.3x10^-5^ nM^-1^ site^-1^ s^-1^ using the mean ± sd of MT length in simulation)*  *[Figs. 2G, H, S2, S3: 4.7x10^-5^ nM^-1^ site^-1^ s^-1^*  *~0.18±0.02 s^-1^nM^-1^]* | ~0.12 s^-1^ nM^-1^ | (Maurer et al., 2014; Song et al., 2020) |
| *k_on, Edge_* | EB1 on-rate constant per protofilament-edge binding site (GTP or GDP) | 1.6x10^-3^ nM^-1^ site^-1^ s^-1^  *(70X increase in on-rate at proto. edges)*  *[Figs. 2G, H, S2, S3: 2.3x10^-3^ nM^-1^ site^-1^ s^-1^*  *(50X increase in on-rate at proto. edges)]* | 50-70X faster than lattice sites | (Reid et al., 2019) |
| *k_off, GDP Lattice_* | EB1 off-rate constant from GDP closed-lattice sites | 1.7-3.3 s^-1^  *[Figs. 2G, H, S2, S3:*  *3.3 s^-1^]* | 3.4±0.2 s^-1^ | (Maurer et al., 2014; Song et al., 2020) |
| *k_off, GTP Lattice_* | EB1 off-rate constant from GTP closed-lattice sites | 0.29 s^-1^ | ~0.38 s^-1^  *(Estimated from*  *k_off, lattice_ above, and reported K_d_ ratio for tip:GDP [=] 285/31 = 9)* | (Maurer et al., 2014; Song et al., 2020) |
| *k_off, GTP Edge_* | EB1 off-rate constant from GTP protofilament-edge sites | 2.9 s^-1^  *(10X increase from GTP closed-lattice sites)* | N/A | ^1^See bond energy justification |

^1^Off-rate from protofilament-edges relative to closed-lattice sites was estimated using bond energy arguments. EB1 bonded to a closed lattice site has 4 bonds, while EB1 bonded to a protofilament-edge site has 2 bonds. Therefore, the off-rates could be related to the energies via:

$\frac{k_{off,edge}}{k_{off,lattice}}\sim\frac{e^{-\frac{2\Delta G}{k_{b}T}}}{e^{-\frac{4\Delta G}{k_{b}T}}}=e^{\frac{\Delta G}{k_{BT}}\left( -2+4 \right)}$ (1)

Thus, a rough estimate of the fold-increase in EB1 off-rates from 4-bond closed lattice sites to 2-bond protofilament edges could be e^2^ = 7. Because an increased off-rate from protofilament edges would decrease the residence time on the protofilament-edges, we used a conservative estimate of a 10-fold increase in EB1 off-rate from GTP-tubulin protofilament edges relatively to GTP-tubulin closed-lattice sites. The simulation was insensitive to the GDP-tubulin protofilament edge off-rate value used (Fig. 1- Fig. supplement 2G-I), because GDP-tubulin edge sites were rarely present in the simulation.
