# Supplementary material for: Rapid binding to protofilament edge sites facilitates tip tracking of EB1 at growing microtubule plus-ends
Source: eLife. 2024 Feb 22;13:e91719. doi: 10.7554/eLife.91719 (PMC10883673; doi:10.7554/eLife.91719)
Supplement: Supplementary file 3. — In addition, tubulin ‘maturation rates’ are compared, which define the EB1 binding zones in the Maurer et al model. [file elife-91719-supp3.docx]

Table S3: Model Parameter Comparison (Maurer et al 2014)

| Model Parameter | Maurer et al 2014 Model | Current Study Model | Comments |
| --- | --- | --- | --- |
| EB1 on-rate (*k_on, edge_*) | N/A | .0016-0.0023 s^-1^ nM^-1^site^-1^ | In current study: On rate to protofilament edges, **per available edge site** (70-fold increased from lattice sites per Reid et al 2019) |
| EB1 on-rate (*k_on, lattice_*) | ~0.12 s^-1^ nM^-1^ | ~0.09-0.18 s^-1^ nM^-1^  *[scaled up from*  *2.3x10^-5^ s^-1^ nM^-1^site^-1^*  *using mean ± sd of MT length in simulation*] | In current study: On rate to closed lattice site, **per available lattice site** |
| EB1 off-rate (*k_off_*) | 3.4±0.2 s^-1^ | 1.7-3.3 s^-1^ | In current study: off-rate from GDP-tubulin closed-lattice sites |
| Tubulin Maturation Rate to allow EB1 binding (*k_1_*) | 1.14 - 10 s^-1^ | N/A | Maturation from EB1-excluded zone to EB1-binding zone. **No EB1 exclusion zone in current study** |
| Tubulin Maturation Rate to allow EB1 unbinding (*k_2_* or *k_hydrolysis_*) | 0.24 – 0.6 s^-1^ | 0.55 s^-1^ | Maurer Model: maturation from EB1 binding zone to EB1 unbinding zone. **In both studies, this is equivalent to the GTP-tubulin hydrolysis rate.** |
